# Critical Care Experience Shapes Respiratory Therapists’ Attitudes toward Death: A Survey Study

**DOI:** 10.3390/healthcare12151533

**Published:** 2024-08-01

**Authors:** Ting-Ling Lee, Jui-O Chen, Nan-Wei Liu, Hui-Chin Chen, Yi-Ling Hsieh, Shih-Feng Liu, Jui-Fang Liu, Hui-Ling Lin

**Affiliations:** 1 Department of Respiratory Therapy, Kaohsiung Chang Gung Memorial Hospital, Kaohsiung 83301, Taiwan; 2 Department of Nursing, Tajen University, Pingtung 90741, Taiwan; 3 Department of English, National Chengchi University, Taipei 11605, Taiwan; 4 Department of Respiratory Care, Chang Gung University of Science and Technology, Chiayi 61363, Taiwan; 5 Department of Respiratory Therapy, Kaohsiung Medical University, Kaohsiung 80701, Taiwan; 6 Division of Pulmonary and Critical Care Medicine, Department of Internal Medicine, Kaohsiung Chang Gung Memorial Hospital, Chang Gung University College of Medicine, Kaohsiung 83301, Taiwan; 7 Chronic Diseases and Health Promotion Research Center, Chang Gung University of Science and Technology, Chiayi 61363, Taiwan; 8 Department of Respiratory Therapy, Chang Gung University, Taoyuan 33302, Taiwan

**Keywords:** respiratory therapists, attitudes toward death, critical care, life and death

## Abstract

Respiratory therapists (RTs) frequently encounter death in their work with critically ill patients. Healthcare providers’ attitudes toward death significantly affect their approach to caring for dying patients; however, there is a lack of knowledge on RTs’ attitudes toward death. This study examines how the work environment and personal characteristics of RTs influence their attitudes toward death. Utilizing the Death Attitude Profile-Revised-Chinese questionnaire, a cross-sectional survey compared non-critical care RTs (non-CCRTs, N = 86) to critical care RTs (CCRTs, N = 85). Non-CCRTs displayed significantly lower scores in overall acceptance of death compared to CCRTs (*p* = 0.015) and a tendency to actively avoid thoughts about death (*p* = 0.005). CCRTs scored higher in “neutral acceptance” (*p* = 0.015), and non-CCRTs exhibited higher scores on items reflecting a negative attitude toward death. RTs with shorter professional tenures showed heightened fear of death and avoidance tendencies. Perception of life and death education correlated with higher “fear of death” and “death avoidance” scores (*p* = 0.001). The findings indicate that CCRTs demonstrate a more neutral acceptance of death. Additionally, experience, sex, mental health status, and life–death education exposure significantly influence RTs’ attitudes toward death.

## 1. Introduction

Death is a neutral aspect of the medical field, making the care of terminally ill patients a crucial concern in healthcare. The handling of a patient’s death can profoundly impact healthcare providers, with some experiencing personal death anxiety and relying on various coping mechanisms [1,2,3]. Healthcare providers’ attitudes toward death significantly affect their approach to caring for dying patients [4].

Death attitudes are frequently studied across various sectors of the healthcare community and social sciences to understand human behavior and improve the experience of facing death. The Death Attitude Profile-Revised, a comprehensive tool for assessing attitudes toward death, is the most commonly used questionnaire to explore beliefs, stresses, anxieties, and influencing factors toward death [5]. The DAP-R encompasses the following five primary dimensions: approach acceptance, fear of death, death avoidance, escape acceptance, and neutral acceptance. Death acceptance refers to an individual’s psychological readiness to confront the end of life. It comprises the following two key elements: a cognitive understanding of mortality and a generally neutral or positive emotional response to this concept. The concept suggests that embracing the reality of death can alleviate fear and anxiety, enabling individuals to lead more purposeful lives. In practical terms, death acceptance is a positive attitude toward death that can serve as a coping mechanism, particularly for healthcare professionals. Fear of death involves a specific and conscious apprehension about the concept of death and dying. This can include concerns about losing one’s identity, uncertainty regarding the afterlife, fear of physical pain or suffering, anxiety over missed opportunities for redemption, and worry about the well-being of loved ones left behind. On the other hand, death avoidance refers to a coping mechanism aimed at regulating and minimizing anxiety related to death, often achieved by avoiding discussions or meditating on the inevitability of death. Escape acceptance occurs when individuals are overwhelmed by suffering and pain, viewing death as the only means of relief. Therefore, escape acceptance reflects a positive attitude toward death, not because death is inherently “good”, but because life is perceived as overwhelmingly “bad”. These dimensions allow for exploration of potential relationships among them. One intriguing possibility is examining how embracing death acceptance might serve as a strategy for managing fear of death. 

Previous research has explored attitudes toward death in various healthcare professionals, including students, physicians, nursing staff, social workers, and palliative care volunteers [6,7,8,9,10]. Tzamakos et al. explored how attitudes toward death correlate with emotional intelligence, personality traits, resilience, and beliefs about justice among midwives. They discovered that a neutral acceptance of death was linked to higher general self-efficacy, which in turn was related to lower personal accomplishment, emotional exhaustion, and job burnout [9,10]. Uzar-Ozcetin et al. investigated how attitudes toward death, rumination, and psychological resilience are interconnected among oncology nurses. They observed that nurses who maintain positive attitudes toward death also tend to develop greater psychological resilience and experience fewer work-related ruminations [11]. 

Within the healthcare landscape, respiratory therapists (RTs) often find themselves at the forefront of managing respiratory diseases, particularly in critical care settings. RTs play a crucial role in the extubation process of terminally ill patients, and statistics show that 10–20% of ICU patients pass away post-withdrawal of life-sustaining treatments, with many having received care from RTs [12]. Experiences related to the care of dying patients influence healthcare professionals’ attitudes and behaviors, impacting job satisfaction and burnout [13,14]. A survey in Taiwan identified factors such as age, years of experience, workplace, religious activities, understanding of palliative care legislation, and relevant in-service education as significant influences on RTs’ attitudes toward caring for dying patients [15]. Despite their vital role, less than 50% of RTs have received education on topics related to patient death or end-of-life issues. Recognizing the importance of understanding healthcare professionals’ perspectives on death, there is a notable gap concerning RTs attitudes toward death. This study examines how the work environment and personal characteristics of RTs influence their attitudes toward death. 

## 2. Materials and Methods

### 2.1. Participants

This study employed a cross-sectional, descriptive survey design conducted from September 2018 to September 2019 in southern Taiwan. It was approved by the Institutional Review Board of Chang Gung Memorial Foundation. The inclusion criteria encompassed registered RTs aged 20 years or older, while part-time or retired RTs were excluded from the study. 

The study utilized convenience sampling, distributing the survey to respiratory therapists during an annual meeting of the Respiratory Therapist Society in southern Taiwan. Participants returned signed informed consent forms and completed questionnaires using prepaid return envelopes addressed to the investigator.

The classification of RTs involved dividing them into two distinct groups: the non-Critical Care RT Group (non-CCRTs) and the Critical Care RT Group (CCRTs), according to their affiliations. Non-CCRTs are engaged in respiratory therapy within non-acute, non-critical care settings, commonly providing services in long-term respiratory care centers, sleep centers, outpatient clinics, pulmonary rehabilitation units, or home settings, dealing predominantly with chronic respiratory conditions. In contrast, CCRTs operate in acute, critical care settings such as intensive care units, emergency rooms, and operating rooms. CCRTs typically possess more extensive exposure and experience dealing with issues related to death.

### 2.2. Instrument

A structured questionnaire was employed for data collection, which included sections on demographic information and the administration of the Chinese version of the Death Attitude Profile-Revised. Demographic characteristics included age, sex, education level, work experience, awareness of physical and mental health, religious beliefs, and attendance at life and death education courses. 

The primary outcomes were assessed using the Death Attitude Profile-Revised, developed by Wong et al. in 1994 and revised in 2015 [5]. The Death Attitude Profile-Revised was translated into Chinese to investigate healthcare providers’ and volunteers’ attitudes toward death [16,17]. We adapted and validated the questionnaire by Wang et al. The process involved two experts with over 30 years of experience in respiratory therapy and one expert with a nursing background and experience in assessing attitudes toward death. These experts were instrumental in refining the Chinese version of the Death Attitude Profile-Revised, which originally contained 32 items. Following their input and a rigorous validation process using Cronbach’s α coefficient (α = 0.853), the survey was carefully revised and finalized to consist of 28 items. This 28-item scale comprises the following five dimensions: fear of death (7 items), death avoidance (5 items), neutral acceptance (5 items), escape acceptance (5 items), and approach acceptance (6 items). The modified Chinese version of the Death Attitude Profile-Revised was structured with a Likert-type scale ranging from 1 (strongly disagree) to 5 (strongly agree). Total scores ranged from 28 to 140, with higher scores indicating a greater inclination toward the corresponding attitude toward death. Subsequently, the survey’s reliability and accuracy were further confirmed through administration to 60 respiratory therapists at Chang Gung Memorial Hospital. The reliability test yielded a Kaiser–Meyer–Olkin measure of 0.836, signifying satisfactory sampling adequacy [18]. 

### 2.3. Data Analysis

Descriptive statistics were used to demonstrate the data, including means, standard deviations (SD), and percentages. An independent samples *t*-test was utilized for statistical analyses between two groups and parameters. The threshold for statistical significance was set at *p* < 0.05. The collected questionnaire data were analyzed using SPSS (version 23.0, IBM Inc., Armonk, NY, USA).

## 3. Results

A total of 220 surveys were distributed, of which 187 respiratory therapists participated in the questionnaire survey for this study, yielding a response rate of 85%. Eleven incomplete responses and five responses from retired individuals were excluded, resulting in 171 completed questionnaires from active RTs being included in the final analysis. Based on their affiliation, 86 responses were from non-CCRTs, while 85 were from CCRTs.

The characteristics of the participants are presented in Table 1. The age of participants ranged from 21 to 60 years, with both groups having an average age between 33 and 35 years. The majority of participants were female, held a university degree, and had less than 10 years of work experience. Most participants perceived their physical and mental health as fair rather than excellent, and a significant portion identified as having no religious beliefs. Both groups had similar distributions regarding participation in formal life and death education courses. The majority in both groups reported that they found life and death education courses helpful in their work. There were no statistically significant differences in demographics between the two groups.

**Figure 1:**
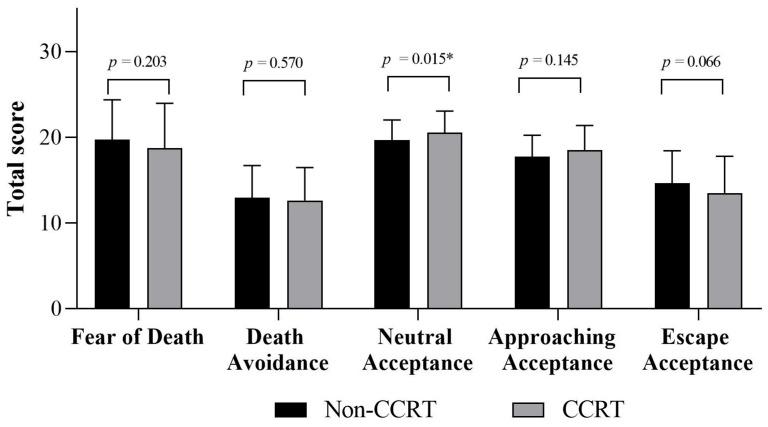
Comparisons on the scores of each dimension between two groups. Abbreviation: CCRT: critical care respiratory therapist; non-CCRT: non-critical care respiratory therapist. * *p* < 0.05.

Table 2 compares the scores of the Death Attitude Profile-Revised between the two groups. The results indicate that the scores for the dimension of “neutral acceptance” are significantly higher in the CCRT group (*p* = 0.015). This is reflected in items such as “Death should be viewed as a neutral, undeniable, and unavoidable event” (*p* = 0.005), “Death is a neutral aspect of life” (*p* = 0.028), and “Death is simply a part of the process of life” (*p* = 0.031). Figure 1 illustrates the comparisons of the total scores between the two groups. Additionally, the non-CCRTs present significantly higher scores on items reflecting a negative attitude toward death, including “The subject of life after death troubles me greatly” (*p* = 0.001) and “I avoid thinking about death altogether” (*p* = 0.035).

To investigate the impact of personal characteristics on death attitudes, we aggregated all the data and conducted *t*-tests to compare differences in subcategories of characteristics. Table 3 outlines the comparisons of each variable on death attitudes. The findings reveal that sex and years of work significantly influence death attitudes among respondents. Female respondents produced a higher total score (*p* = 0.023), primarily driven by the dimension of “approaching acceptance” (*p* = 0.004). Years of working experience (*p* = 0.043) also had an impact on death attitudes, particularly on “fear of death” (*p* = 0.037) and “death avoidance” (*p* = 0.005); greater experience correlated with lower scores in these dimensions.

## 4. Discussion

Respondents who perceived their mental health condition as excellent had lower scores on “fear of death” (*p* = 0.024) and higher scores on “neutral acceptance” (*p* = 0.048). Experience of life and death education through continuing education resulted in higher scores in “fear of death” (*p* = 0.036). The perception of the impact of life and death education yielded higher scores in “fear of death” and “death avoidance” (*p* = 0.001). However, the physical condition and religion of the participants did not influence death attitudes.

Respiratory therapists frequently find themselves in environments where they routinely encounter life-and-death situations, particularly within critical care units. In these settings, RTs have frequented direct interactions with terminally ill patients, allowing them to observe and engage in the dying process. Previous research has emphasized the crucial link between healthcare professionals’ attitudes towards death and the quality of care they provide [19,20]. This study sheds further light on the variations in attitudes towards death between the CCRT and non-CCRT groups. Specifically, in terms of the attitude of neutral acceptance towards death, CCRTs tend to embrace death more readily. In contrast, the non-CCRT group demonstrates lower levels of neutral acceptance towards death and higher levels of fear of death. Additionally, our study reveals that personal experiences and characteristics significantly influence RTs’ attitudes towards death. Participation in life and death education programs was associated with reduced fear of death and an increased acceptance of death as a neutral aspect of life. This emphasizes the importance of targeted educational interventions in shaping healthcare professionals’ perspectives on death.

### 4.1. The Impact of Experiences in Critical Care on the Attitudes toward Death among RTs

Most RTs are employed in acute care hospitals, playing a crucial role in managing mechanical ventilation for critically ill patients. Regrettably, despite the provision of mechanical ventilation and other advanced life-sustaining measures, some patients may not survive. RTs frequently participate in end-of-life patient care, which can encompass the discontinuation of mechanical ventilation. While most studies have focused on death anxiety among RTs [21,22,23], our measurement included a broader range of cognitive and emotional responses, termed “death attitudes”. 

Initially, we categorized RTs into two groups based on their work settings. Non-CCRTs typically deliver respiratory therapy services in long-term care centers, outpatient clinics, or home settings, where they have fewer experiences directly related to death in their professional duties. On the other hand, CCRTs operate in critical care environments where they frequently engage with profound exposure and experience issues associated with deaths or the dying process. This includes managing life support equipment, participating in emergency interventions, engaging in discussions with patients and their families about treatment plans and end-of-life choices, or performing terminal weaning from mechanical ventilators and extubation.

Our findings revealed a significant difference in the perception of “neutral acceptance” between the two groups. CCRTs tended to view death as a neutral process of life and acknowledged it as an inevitable and unavoidable event. The majority of respondents reported that death was an inherent aspect of the life process. Previous studies on nursing students have also reported high scores in the “neutral acceptance” dimension, indicating that although participants may fear death, they recognize it as a neutral part of life’s cycle among different healthcare providers [4,24,25]. 

Work settings and experiences play a significant role in shaping individuals’ coping mechanisms with death, reflecting in their attitudes toward death [26,27]. CCRTs, who regularly interact with critically ill or dying patients, often confront death and dying situations, leading to a more positive “neutral acceptance” attitude toward death. Most CCRTs reported lower scores on the “fear of death” dimension, indicating a lesser degree of fear regarding death. Conversely, non-CCRTs exhibited a higher degree of negative thoughts, particularly expressing greater concern about life after death. Since non-CCRTs have fewer experiences with the dying process and deaths in their daily practice, they tend to experience heightened anxiety when faced with death-related situations. Previous research has suggested that healthcare providers’ attitudes toward death may gradually change as they gain more experience dealing with death and dying situations [28]. 

Research has shown that cultivating a positive attitude toward death among healthcare professionals, including nurses, can help reduce their fear of death [13,29]. This positive attitude may stem from a deeper understanding and acceptance of death as a neutral part of life, ultimately alleviating anxiety and fear surrounding the topic. By fostering a healthy perspective on death, healthcare providers can approach end-of-life care with greater compassion, empathy, and resilience, enhancing the quality of care provided to terminally ill patients and their families [3]. The longer healthcare providers work and the more frequently they care for critically ill patients, the less fear of death they tend to have, as their experiences accumulate with seniority [13,30]. 

The total scores for “death avoidance” were low in both groups, with non-CCRTs scoring significantly lower on “I avoid thinking about death altogether”. The non-CCRT group, compared to the CCRT group, had less exposure to the process of patient death or the dying process, leading to stronger avoidance tendencies when facing death. This finding aligns with Asadpour et al.’s survey of 308 medical students, suggesting that individuals with more experience in dealing with death tend to exhibit fewer attitudes of avoidance toward it [31]. Similarly, this finding aligns with the results of Guo’s investigation into the attitudes toward death among oncology nursing staff, where individuals with more experience tended to exhibit less avoidance in confronting death [13].

Finally, our study showed that the vast majority of respondents did not exhibit a clear tendency toward “approaching acceptance” of death. However, within the CCRT group, some respondents believed that due to their religious beliefs or commitments, they anticipate a better afterlife, which makes them less fearful of death. Specifically, in response to questions regarding “Death is a union with God (the divine, Buddha, etc.) and eternal bliss” and “Death will bring forth a new and glorious life”, the scores among the CCRT group were significantly higher than those in the non-CCRT group. This finding differs from the argument proposed by Peterson et al. that religious beliefs can help healthcare professionals accept death and alleviate the distress caused by death [32]. In contrast, Barnett found that religious commitment correlated with “fear of death”, “approach acceptance”, and “escape acceptance” [1]. Our study found no direct correlation between religious beliefs and attitudes toward death among the two groups, which warrants further exploration into the underlying reasons.

### 4.2. The Impact of Personal Characteristics on the Attitudes toward Death among RTs

Our exploration of the associations between personal experiences and characteristics and death attitudes revealed that individuals who had received death education courses and perceived them as helpful for their work were less likely to experience fear or avoidance toward death and more likely to accept death as a natural aspect of life. This finding aligns with studies by Ceyhan et al. and Xu et al., asserting that training courses on end-of-life care can empower healthcare professionals with knowledge, social–psychological skills, and cultural sensitivity, fostering positive attitudes and reducing anxiety [25,33]. Consistent with the findings of Dong et al., our study indicates that nurses who underwent death education training were more likely to exhibit a higher level of acceptance of death as a neutral aspect of life [19]. Additionally, Zhao et al. suggested that nursing managers should pay special attention to nurses with shorter tenures, assisting them in death education to overcome fear of death and confront work related to critically ill patients and death [34].

Our study also revealed that scores of RTs who had not received death education courses were significantly higher than those who had. Regarding the fear of death dimension, healthcare professionals who had received training on death-related issues were better able to accept death. These findings underscore the importance of healthcare professionals receiving education and training on death-related issues, especially for newcomers in healthcare [6,7,35,36]. 

Individuals with excellent self-perceived psychological health status exhibited lower levels of fear of death and higher levels of neutral acceptance of death compared to those with ordinary psychological health status. This result aligns with previous research showing a close correlation between nurses’ attitude toward death and their subjective well-being [37]. Individuals with excellent psychological health status are less likely to experience fear of death and are more able to accept death as a neutral process of life and as a part of life.

Our study found that RTs with shorter tenures exhibited higher levels of fear of death and avoidance tendencies. Factors such as gender, psychological health status, and the degree of acceptance of death education also influenced RTs’ attitudes toward death. In response to these factors, medical institutions should offer personalized support and training based on individual characteristics and needs to assist these RTs in handling death-related situations more confidently.

In medical schools or workplace training programs, there should be more discussions on death-related topics, and methods such as scenario-based simulations could be utilized to enhance RTs’ ability to cope with patient deaths. Additionally, there should be enhanced grief counseling and counseling courses to provide the necessary support and assistance for RTs when facing death [38]. Furthermore, peer debriefing activities can promote mutual support and growth among RTs, enabling them to better address the challenges they may encounter in their work. Employing scenario-based simulation teaching methods can enhance RTs’ coping abilities when facing patient deaths. Additionally, increasing grief counseling and counseling courses can serve as a foundation for strengthening RTs’ ability to cope with death. Utilizing debriefing activities can foster peer support and growth among RTs.

### 4.3. Cultureal Considerations on the Attitudes toward Death among RTs

This study was conducted in the southern region of Taiwan, and demographic factors and local beliefs may impact individuals’ attitudes toward death and coping mechanisms. Previous studies have reported that nursing staff may develop different attitudes toward death based on their socio-cultural characteristics, learning, and experiences. These attitudes can further have a positive or negative impact on their perspectives on death and their nursing work [39,40]. Furthermore, in Chinese culture, death is often viewed as a taboo subject. It is commonly avoided in open discussions, even within families. This tendency is observed to be more pronounced among males than females. Males are taught to be tough, while females are encouraged to express their emotions. Healthcare providers, in particular, may suppress their own emotions and grief when witnessing patients’ deaths [15,41,42]. Our results demonstrate a significantly lower score on the “Approaching acceptance” domain among males. Further education could be designed to enhance coping mechanisms and encourage the free expression of emotions across different sexes and genders.

### 4.4. Limitations

Despite its contributions, our study has certain limitations. It solely relied on questionnaire surveys to assess RTs’ attitudes toward death. Future research should consider collecting qualitative data to gain a more comprehensive understanding of RTs’ perspectives on end-of-life care and attitudes toward death. As a result, participants’ survey responses may not fully reflect their true opinions and feelings on the matter. Therefore, further qualitative surveys may be warranted to gain a deeper understanding of their perspectives on death attitude and end-of-life care. Additionally, this study was conducted prior to the COVID-19 pandemic, during a time when RTs cared for patients on the frontlines and experienced higher mortality rates. The COVID-19 pandemic has heightened RTs’ exposure to critically ill patients, presenting more opportunities for them to confront end-of-life situations. D’Alessandro-Lowe et al. reported increased moral distress, symptoms of post-traumatic stress disorder, anxiety, and dissociation among Canadian respiratory therapists. These therapists worked under limited resources, facilitating calls between dying patients and their families during the pandemic [43]. The prolonged exposure to this tragedy may have influenced their perceptions and attitudes toward death. Further research is needed to examine how their perceptions and attitudes may have changed before and after the pandemic. 

## 5. Conclusions

Death is an inevitable part of life, and RTs stand at the forefront of critical care, frequently encountering patient deaths and the dying process. Our study indicates that the CCRT group tends to exhibit a more neutral acceptance attitude toward death compared to the non-CCRT group. Furthermore, it reveals that individuals with more exposure to death experiences and those who have received death education exhibit more positive attitudes toward death. Encouraging the incorporation of discussions on death-related topics in both school education curriculums and hospital continuing education programs for RTs is recommended. Additionally, further education, including open discussions on cultural backgrounds and death education across different genders, could positively influence healthcare providers’ attitudes toward death. This proactive approach can enhance RTs’ ability to navigate and cope with the challenging aspects of their profession, fostering a more positive and accepting outlook on the inevitable aspect of life that is death. For future research, we recommend further collecting qualitative data to gain a deeper understanding of the formation of RTs’ attitudes toward death and the influencing factors. Additionally, assessing the effectiveness of corresponding intervention measures would be beneficial. This approach will help enhance RTs’ acceptance of death, thereby further improving respiratory care for terminally ill patients.

## Figures and Tables

**Table 1:** Demographics of participants.

Variables	Non-CCRT (N = 86)	CCRT (N = 85)	*p*-Value
Age	35.18 ± 7.77	33.72 ± 7.20	0.206
Sex			0.116
Male; n (%)	20 (23.3)	29 (34.1)	
Female; n (%)	66 (76.7)	56 (65.9)	
Degree			0.166
Associated; n (%)	16 (18.6)	8 (9.4)	
Bachelor; n (%)	59 (68.6)	61 (71.8)	
Masters; n (%)	11 (12.7)	16 (18.8)	
Work experience			0.536
<10 years; n (%)	56 (66.8)	49 (57.6)	
>10 years; n (%)	30 (33.2)	36 (42.4)	
Self-perceived Physical Health Condition			0.307
Excellent; n (%)	10 (11.6)	21 (20.0)	
Good; n (%)	76 (88.4)	64 (80.0)	
Self-perceived Mental Health Condition			0.123
Excellent; n (%)	11 (12.8)	17 (24.7)	
Good; n (%)	75 (87.2)	68 (75.3)	
Religious Belief			0.645
Yes; n (%)	36 (41.9)	38 (45.1)	
No; n (%)	50 (58.1)	47 (54.9)	
Life and Death Education			0.479
Formal Course; n (%)	54 (62.8)	48 (56.5)	
Informal Course; n (%)	32 (37.2)	37 (43.5)	
Impact of Life and Death Education Courses on Work			0.134
Yes; n (%)	80 (93.0)	72 (84.7)	
No; n (%)	6 (7.0)	13 (15.3)	

Abbreviation: CCRT: critical care respiratory therapist; non-CCRT: non-critical care respiratory therapist.

**Table 2:** Comparisons of perspectives on death attitudes.

Items	Non-CCRT (N = 86)	CCRT (N = 85)	*p*-Value
Total score	84.7 ± 11.28	85.09 ± 11.54	0.737
Dimension: Fear of Death	19.70 ± 4.67	18.73 ± 5.23	0.203
Death is no doubt a grim experience.	3.12 ± 0.87	3.13 ± 0.91	0.923
The prospects of my own death arouse anxiety in me.	3.26 ± 0.90	3.13 ± 1.00	0.385
I am disturbed by the finality of death.	2.38 ± 0.87	2.33 ± 0.96	0.698
I have an intense fear of death.	2.60 ± 0.86	2.51 ± 0.98	0.485
The subject of life after death troubles me greatly.	2.86 ± 0.92	2.40 ± 0.90	0.001 **
The fact that death will mean the end of everything as I know it frightens me.	2.57 ± 0.93	2.45 ± 1.03	0.414
The uncertainty of not knowing what happens after death worries me.	2.91 ± 0.82	2.79 ± 1.04	0.408
Dimension: Death Avoidance	12.95 ± 3.76	12.62 ± 3.83	0.570
I avoid death thoughts at all costs.	2.76 ± 0.93	2.84 ± 1.06	0.602
Whenever the thought of death enters my mind, I try to push it away.	2.65 ± 0.93	2.53 ± 0.95	0.397
I always try not to think about death.	2.59 ± 0.91	2.52 ± 0.85	0.578
I avoid thinking about death altogether.	2.47 ± 0.92	2.18 ± 0.86	0.035 *
I try to have nothing to do with the subject of death.	2.49 ± 0.84	2.56 ± 0.92	0.571
Dimension: Neutral Acceptance	19.64 ± 2.36	20.55 ± 2.51	0.015 *
Death should be viewed as a neutral, undeniable, and unavoidable event.	4.22 ± 0.77	4.52 ± 0.57	0.005 *
Death is a neutral aspect of life.	4.19 ± 0.62	4.40 ± 0.64	0.028 *
I would neither fear death nor welcome it.	3.35 ± 0.73	3.51 ± 0.93	0.222
Death is simply a part of the process of life.	4.05 ± 0.69	4.28 ± 0.72	0.031 *
Death is neither good nor bad.	3.85 ± 0.74	3.85 ± 0.81	0.988
Dimension: Approaching Acceptance	17.76 ± 2.46	18.49 ± 2.88	0.145
I believe that I will be in heaven after I die.	3.17 ± 0.72	3.07 ± 0.83	0.383
Death is an entrance to a place of ultimate satisfaction.	2.86 ± 0.92	2.79 ± 0.79	0.583
I believe that heaven will be a much better place than this world.	3.03 ± 0.98	3.19 ± 0.91	0.288
Death is a union with God and eternal bliss.	2.94 ± 0.89	3.19 ± 0.73	0.049 *
Death brings a promise of a new and glorious life.	2.77 ± 0.73	3.11 ± 0.77	0.012 *
I see death as a passage to an eternal and blessed place.	2.98 ± 0.91	3.15 ± 1.21	0.287
Dimension: Escape Acceptance	14.63 ± 3.79	13.48 ± 4.30	0.066
Death will bring an end to all my troubles.	3.03 ± 1.12	2.87 ± 1.12	0.339
Death provides an escape from this terrible world.	2.73 ± 0.93	2.49 ± 0.98	0.104
Death is deliverance from pain and suffering.	3.01 ± 0.98	2.73 ± 0.96	0.058
I view death as a relief from earthly suffering.	2.83 ± 0.94	2.61 ± 1.00	0.151
I see death as a relief from the burden of this life.	3.02 ± 0.88	2.78 ± 1.02	0.091

Abbreviation: CCRT: critical care respiratory therapist; non-CCRT: non-critical care respiratory therapist. * *p* < 0.05; ** *p* < 0.001.

**Table 3:** Influence of personal characteristics toward death attitude.

Dimensions		Fear of Death		Death Avoidance		Neutral Acceptance		Approaching Acceptance		Escape Acceptance		Total Score	
Variables	n	Mean ± SD	*p*-Value	Mean ± SD	*p*-Value	Mean ± SD	*p*-Value	Mean ± SD	*p*-Value	Mean ± SD	*p*-Value	Mean ± SD	*p*-Value
Sex			0.308		0.485		0.858		0.004 *		0.064		0.023 *
Male	n = 49	18.61 ± 5.24		12.33 ± 4.31		20.09 ± 2.43		16.78 ± 3.81		12.98 ± 4.51		80.78 ± 12.07	
Female	n = 122	19.44 ± 4.86		12.96 ± 3.58		20.10 ± 2.50		18.62 ± 2.97		14.46 ± 3.85		85.58 ± 10.87	
Work experience			0.037 *		0.005 **		0.443		0.522		0.375		0.043 *
<10 years	n = 56	20.38 ± 4.53		14.00 ± 3.28		19.83 ± 2.42		18.13 ± 2.71		13.72 ± 4.37		86.06 ± 10.40	
>10 years	n = 115	18.70 ± 5.10		12.27 ± 3.91		20.19 ± 2.51		18.16 ± 3.55		14.22 ± 3.95		83.52 ± 11.83	
Self-perceived mental health condition			0.024 *		0.365		0.048 *		0.396		0.204		0.091
Excellent	n = 32	17.56 ± 5.05		12.38 ± 3.15		20.81 ± 2.32		17.97 ± 3.40		13.25 ± 4.76		81.97 ± 11.94	
Good	n = 138	19.61 ± 4.90		12.88 ± 3.94		19.93 ± 2.50		18.17 ± 3.30		14.19 ± 3.87		84.80 ± 11.26	
Self-perceived physical health condition			0.094		0.677		0.341		0.908		0.746		
Excellent	n = 27	18.00 ± 4.85		13.22 ± 3.57	.	20.48 ± 2.52		18.37 ± 3.40		14.48 ± 4.09		84.56 ± 11.54	0.802
Good	n = 144	19.49 ± 4.95		12.77 ± 3.82		19.98 ± 2.45		18.05 ± 3.31		14.00 ± 4.11		84.28 ± 11.43	
Religious belief			0.515		0.052		0.639		0.892		0.876		0.792
Yes	n = 96	18.91 ± 5.09		13.19 ± 3.81		19.98 ± 2.65		18.02 ± 3.64		14.16 ± 4.24		84.25 ± 11.26	
No	n = 75	19.69 ± 4.79		12.31 ± 3.73		20.29 ± 2.22		18.27 ± 2.86		13.96 ± 3.91		84.52 ± 11.56	
Life and death education experience			0.036 *		0.320		0.050		0.432		0.189		0.158
Formal Course	n = 102	14.87 ± 4.23		12.29 ± 3.41		20.87 ± 2.33		16.97 ± 4.06		13.06 ± 4.72		78.06 ± 12.52	
Continue education	n = 69	16.67 ± 4.28		13.01 ± 3.70		19.93 ± 2.44		17.58 ± 3.88		14.11 ± 3.81		81.30 ± 11.23	
Perception impact of life and death education courses			<0.001 **		0.001 **		0.252		0.247		0.881		0.162
Yes	152	15.95 ± 4.48		12.50 ± 3.79		20.19 ± 2.48		17.75 ± 4.17		14.28 ± 3.93		80.66 ± 12.20	
No	19	20.19 ± 3.47		15.81 ± 2.81		19.44 ± 2.37		17.63 ± 3.03		14.13 ± 3.72		87.19 ± 8.18	

Abbreviation: CCRT: critical care respiratory therapist; non-CCRT: non-critical care respiratory therapist. * *p* < 0.05; ** *p* < 0.001.

## Data Availability

The data that support the findings of this study are available from the corresponding author (H.-L.L.) upon reasonable request.
